# The effect of roselle leaf (*Hibiscus sabdariffa L*.) extract gel on wound healing

**DOI:** 10.25122/jml-2021-0425

**Published:** 2022-10

**Authors:** Puspita Sari Rambe, Imam Budi Putra, Ariyati Yosi

**Affiliations:** 1 Department of Dermatology and Venereology, Faculty of Medicine, Universitas Sumatera Utara, Medan, Indonesia

**Keywords:** roselle, leaf, *Hibiscus sabdariffa L*., wound healing

## Abstract

Roselle (*Hibiscus sabdariffa L*.) belongs to the genus *Hibiscus* with proven anti-inflammatory, antioxidant, and antimicrobial properties. Scientific evidence associated roselle content with bioactive compounds, such as phenolic acids, flavonoids, and anthocyanin. Most studies focused on their petals, while research on leaf extract on wound healing has never been done. This study aimed to assess the effect of roselle leaf extract on wound healing in rats. This was an experimental laboratory study with a posttest-only control group design. There were 30 rats divided into 5 groups: negative control, 5%, 10%, and 15% roselle leaf extract, and positive control (bioplacenton). The parameters assessed in this research were wound size and histological assessment. The data were analyzed using ANOVA. P<0.05 was considered statistically significant. Wound healing percentage and epithelial thickness in the 15% group were the largest (84.17%; 64.69 µm). The lowest value was recorded in placebo (64%; 36.33 µm). Meanwhile, wound healing percentage and epithelial thickness of rats in the 5% and 10% groups were 68.53%, 43,57 µm, and 78.11%, 56.49 µm, respectively. Finally, positive control had a 77.44% wound healing percentage and 49.7 µm epithelial thickness. There were no significant differences in wound healing and epithelial thickness among the groups. Roselle leaf extract at 15% concentration showed greater wound healing properties based on clinical and histological assessment. Although there were no statistically significant differences, roselle leaf showed an opportunity to be further investigated as a potential wound healing therapy.

## INTRODUCTION

Wound healing is a complex process involving many types of interactions among cells and biochemical mediators. The process triggers a series of sequential and overlapping events categorized into several phases [1]. Those phases are the hemostasis or coagulation phase, inflammation phase (day 1–3), proliferation and repair phase (day 4–21), and finally, remodeling phase (day 21–365) [2]. Synthesis of Interleukin-1b (IL-1b) occurs after 12–24 hours of wound infliction, and basal level is achieved after completing the proliferative stage. In addition, the Inflammatory phase begins right after the inflammatory chemokines are activated [3].

The IL-1β is a potent inflammatory agent which aggregates the neutrophils into the site of infection [4]. Both macrophages and T lymphocytes play an important role in synthesizing the tumour necrosis factor-alpha (TNF-α); its level can increase due to inflammation and infection conditions [3].

TNF-α acts as host defense, performs phagocytosis and clears debris and dead cells [5]. Furthermore, macrophages, which are activated by IL-4 and IL-13, reduce inflammation by releasing anti-inflammatory cytokines such as IL-10 [1], inhibiting the production of IL-1β, IL-6, and TNF-α [5].

After achieving a balanced inflammatory response, the healing cascade will then enter the proliferative phase, which is a complex process consisting of angiogenesis, granulation tissue formation, collagen deposition, epithelialization, and wound retraction that occur simultaneously [6]. Vascular endothelial growth factor (VEGF) induces angiogenesis and triggers cell migration and proliferation. The proliferative phase can be increased significantly by tumour growth factor-b (TGF-b) since it can improve proliferation, collagen production, differentiation of fibroblasts, and wound contraction [3].

Meanwhile, wound disorders influence the increase of oxidative stress, leading to the high production of reactive oxygen species (ROS) in the wound area. The inflammatory phase can be shortened by applying antibacterial and antioxidant agents. Also, it can accelerate the healing process and promote the proliferative phase [3].

Natural remedies for wound healing have improved significantly. Natural remedies in the form of herbal treatment produce wound healing products that are considered to have far greater drug safety than chemical drugs at more affordable prices [7]. It has been known for many years that medicinal plants and extracts have been used by people to promote wound healing. This is because such plants may contribute to wound healing due to their antioxidant properties and by enhancing angiogenesis, collagen synthesis, and preventing inflammation [8].

Roselle (*Hibiscus sabdariffa L*.) leaves contain major subclasses of flavonoids, such as rutin, quercetin, and kaempferol and their derivatives [9, 10]. In addition, roselle leaves (*H. sabdariffa L*.) have also been identified to contain neochlorogenic acid, chlorogenic acid, cryptochlorogenic acid [11], as well as protocatechuic acid, and sitosterol-β-D-galactoside [9].

Roselle leaves contain a higher concentration of polyphenolic compounds, especially chlorogenic acid, quercetin, and kaempferol, contributing to antioxidant capacity and anti-inflammatory activity [12]. It also functions as an antimicrobial and potent free radical scavenger against reactive oxygen species (ROS) [13]. In addition, anthocyanins in roselle leaves play an important role in the oxidative stress response because of their ability to eliminate ROS [10]. Anthocyanins have also been investigated to have anti-inflammatory potential by reducing the levels of inflammatory mediators [14].

The leaves are the most extensive parts of the roselle plants, but they are underutilized if compared to the petals [12]. Until now, there has been no research about the effect of roselle leaf extract on wound healing. Available research is still limited to the use of roselle petals. Therefore, this research was conducted to assess the effect of roselle leaf extract on the wound healing process.

## Material and Methods

### Roselle leaf extract preparation

*Hibiscus sabdariffa L*. leaves were collected from Tanjung Morawa, North Sumatra, Indonesia and validated by Herbarium Medanese, Universitas Sumatera Utara, Indonesia. The leaves were dried in a dryer at 40–50℃, and 500 g of the simplicia powder was inserted into a container prepared for maceration. Next, 5000 ml of 96% ethanol was added to the container. The mixture was stirred for 6 hours, covered, and left for 18 hours away from lights. Next, the mixture was filtered, where the sediment was collected and soaked again in ethanol 3 times. The macerate was allowed to stand for 24 hours to ensure complete extraction of all bioactive components. Finally, the mixture was evaporated using a rotary evaporator at 40–50℃ until a thick extract was obtained.

### Gel formulation

Three types of gel were formulated from the roselle leaf extracts: 5%, 10%, and 15%. Gel base from carbopol, glycerin, propylene glycol, TEA, nipagin and distilled water was used as the negative control. Bioplacenton gel (placenta extract 10%, neomycin sulfate 0,5%, PT. Kalbe Farma TBK, Bekasi, Indonesia) was used as standard medicine to compare wound healing potential from the extract.

### Qualitative phytochemical evaluation

Phytochemical identification test showed that roselle leaf extract contained alkaloids, flavonoids, glycosides, saponins, tannins, and triterpenoids/steroids.

### Experimental animals

The experimental animals used in this research were 30 white Wistar male rats (Rattus novergicus) with a body weight between 150–200 gr. They were divided into 5 groups, such as negative control, 5%, 10%, and 15% roselle leaf extract, and positive control (bioplacenton) groups, and put in separate cages according to the given treatment. The rats were given food and drinks ad libitum and quarantined for 7 days to adapt to the new environment.

### Wound models

The rats were anesthetized using ketamine 50 mg/ml intraperitoneally as much as 0.2 ml, and the fur was shaved at 3×3 cm on the back area. The excision wound was 15 mm in diameter and 2 mm in depth. They were divided into 5 groups of 6. The negative control group received wound treatment with the gel base, the positive control group were given bioplacenton gel for the wound, while groups with roselle leaf gel extract were given 5%, 10%, and 15% roselle leaf extract gel accordingly. All gels were applied twice a day for 14 days.

### Measurement of wound area

Wound diameter measurement was carried out on days 1, 7, and 14 using a digital caliper. The percentage of wound healing was obtained by the following calculation:


$$\%\text{ wound healing = }\frac{Wound\text{ size diameter on D0-Wound size diameter on D14}}{Wound\text{ size diameter on D0}}\times 100\%$$

### Histological assessment

On day 14, the excision on the wound healing area was carried out to assess the thickness of epithelialization. The tissue was fixed using a 10% formalin structure and prepared for histopathological preparation. The sample was observed using a light microscope (Olympus BX 53 Microscope) at 100x and 400x magnification connected to the OptiLab Microscope camera. The observation was carried out on a display computer monitor.

### Statistical analysis

Data in this research was analyzed by ANOVA test and presented as mean±standard deviation (SD), with p<0.05 considered significant.

## Results

Table 1 shows the highest wound contraction ability in the 15% roselle leaf extract group, followed by the 10% roselle leaf extract group, with an average of 2.38 mm and 3.28 mm, respectively. This contraction ability was higher compared to placebo (5.4 mm) and positive control (3.38 mm) groups.

**Table 1:** Wound size in rats based on treatment group.

Treatment Group	n	Wound size, average (SD), mm				p
		D0	D1	D7	D14	
**Placebo**	6	15	14.49 (0.27)	9.63 (1.20)	5.40 (1.23)	0.002
**Bioplacenton**	6	15	14.65 (0.11)	10.08 (1.47)	3.38 (2.54)	0.005
**Roselle leaf 5%**	5	15	14.57 (0.29)	9.45 (2.45)	4.72 (1.77)	0.018
**Roselle leaf 10%**	6	15	14.58 (0.32)	8.88 (1.44)	3.28 (2.80)	0.013
**Roselle leaf 15%**	6	15	14.69 (0.22)	9.47 (1.02)	2.38 (2.22)	0.002

The wound healing percentage value between day 0 (D0) and 14 (D14) is shown in Table 2. The highest wound healing percentage was shown in the 15% roselle leaf extract group, with an average of 84.17% wound healed. This result was followed by the 10% roselle leaf extract group with an average of 78.11% wound healed. Meanwhile, the lowest wound healing percentage was observed in placebo group rats, with an average of 64% wound healed. ANOVA test did not show a significant difference in the wound healing percentage among the groups (p=0.167). Although no statistically significant differences were found, clinically, almost 100% wound healing on D14 was observed in 2 rats and 1 rat in group 15% and 10% roselle leaf extract gel, respectively.

**Table 2:** Wound healing percentage based on treatment group.

Intervention Group	n	Wound Healing Percentage, %		p
		Average (SD)	Median (Min–Max)	
**Placebo**	6	64 (8.17)	62.17 (55.67–78)	0.167
**Bioplacenton**	6	77.44 (16.95)	84 (48.67–92.33)	
**Roselle leaf 5%**	5	68.53 (11.8)	66 (58–86.67)	
**Roselle leaf 10%**	6	78.11 (18.69)	83.67 (50.33–100)	
**Roselle leaf 15%**	6	84.17 (14.83)	84.17 (62.33–100)	

**Figure 1:**
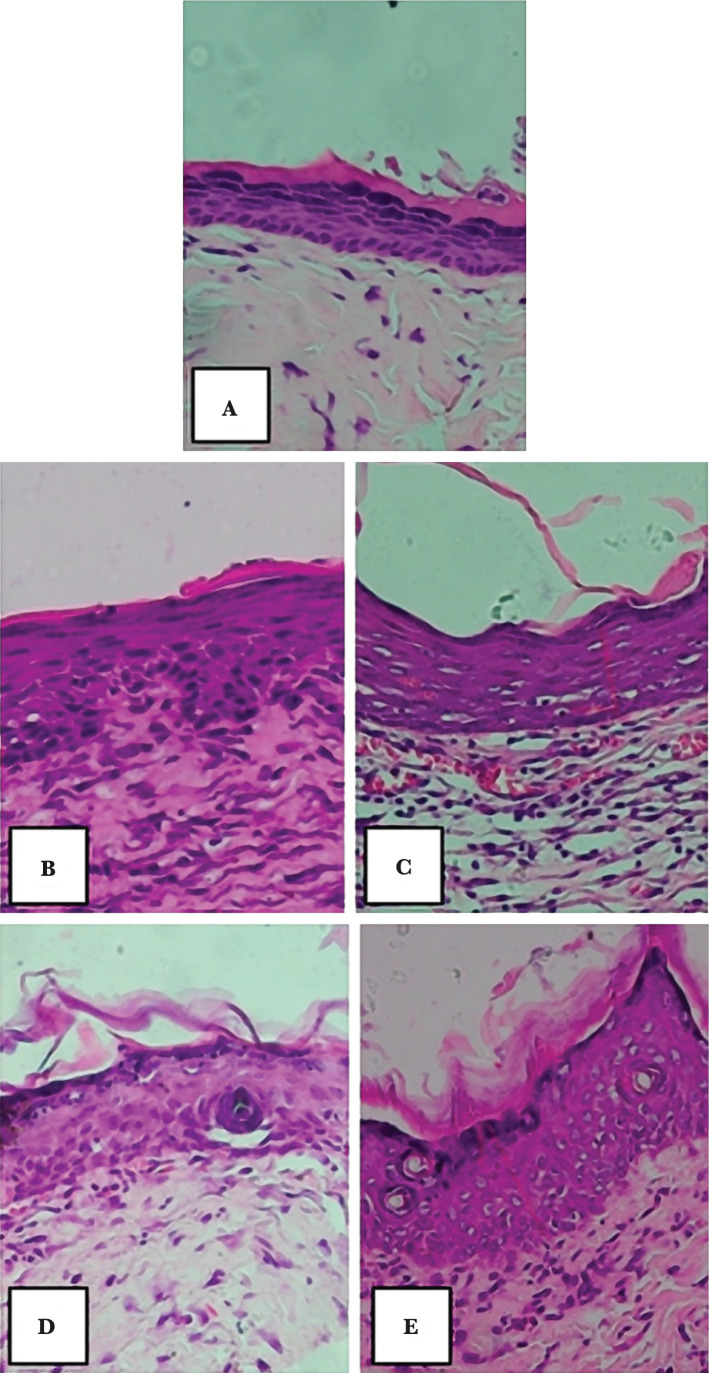
Re-epithelialization of excision wound on day 14 using hematoxylin and eosin staining, with 100x magnification. Remarks: A – placebo; B – bioplacenton gel; C – 5% roselle leaf extract gel; D – 10% roselle leaf extract gel; E – 15% roselle leaf extract gel.

Epithelial thickness was measured after 14 days of treatment using hematoxylin and eosin (H&E) staining. The histological observation result for epithelial thickness was measured from the stratum basale layer to the stratum corneum layer (Figure 1). In Figure 1, it can be seen the re-epithelialization of the excision wound in the group who were given placebo (Figure 1 A), bioplacenton gel (Figure 1 B), roselle leaf extract gel in the amount of 5%, 10%, and 15% (Figure 1 C–E).

Table 3 shows the average epithelial thickness, where the 15% roselle leaf extract gel group had an average thickness of 64.69 µm, followed by the 10% roselle leaf extract gel group with an average of 56.49 µm. The lowest epithelial thickness was observed in the placebo group, with an average of 36.33 µm. In the bioplacenton gel group, the average epithelial thickness was 49.70 µm. The ANOVA test did not show significant differences in the epithelial thickness among the groups (p=0.330). Although there were no statistically significant differences, histological assessment showed that the 15% roselle leaf extract gel group had the greatest epithelial thickness, while the least epithelial thickness growth was observed in the placebo group. Consequently, the 15% roselle leaf extract gel treatment had a faster re-epithelialization process in wound healing.

**Table 3:** Epithelial thickness based on treatment group.

Intervention Group	n	Epithelial thickness, µm		p
		Average (SD)	Median (Min–Max)	
**Placebo**	6	36.33 (17.25)	29.4 (20.59–64.78)	0.330
**Bioplacenton**	6	49.70 (21.14)	42.69 (22.75–78)	
**Roselle leaf 5%**	5	43.57 (11.11)	41.26 (31.72–61.79)	
**Roselle leaf 10%**	6	56.49 (25.78)	47.43 (28.67–91.81)	
**Roselle leaf 15%**	6	64.69 (36.40)	54.20 (27.03–126.65)	

## Discussion

A prolonged-inflammatory response (chronic inflammation) may inhibit the wound healing process. Therefore, improvement in inflammatory responses can represent a therapeutic target for wounds [1]. Roselle leaves have been known for their phytochemical contents, such as anthocyanins and quercetin, that have anti-inflammatory effects. Anthocyanins were studied to have anti-inflammatory potential by *in vivo* reduction of inflammatory mediators like interleukin (IL)-6 and tumor necrosis factor-alpha (TNF-α) [14]. Quercetin is also a potent antioxidant with anti-inflammatory properties. This phytochemical can suppress inflammation by decreasing major pro-inflammatory cytokines, such as TNF-α and IL-1ß [15]. In addition, the compound was shown to increase IL-10 content [16], which can limit inflammatory responses and stimulate wounds to enter the proliferation phase [17]. Therefore, the phytochemical content can suppress the inflammatory phase, accelerating wound repair, as shown in the roselle leaf extract group. The highest wound contraction was found in the 15% roselle group, followed by the 10% roselle group. This contraction ability is higher compared to both the placebo group and the positive control group.

As a result of the inflammatory process, reactive oxygen species (ROS) continuously produce and inhibit the wound healing process due to their damaging effects on cells and tissues [18]. Antioxidants can significantly improve tissue damage and accelerate healing [15]. Rutin (quercetin-3-rutinoside) is one of the major flavonoids found in roselle leaves [10] that can eliminate free radicals, including hydroxyl, superoxide, and peroxyl radicals [18]. Similarly, chlorogenic acids and flavonoids found in roselle leaves [11] have antioxidant effects by inhibiting the production of free radicals and facilitating antioxidant enzymes [19]. The presence of antioxidants in roselle leaves can accelerate the wound healing process, as seen in the 15% roselle leaf extract group, which shows the highest wound contraction, followed by the 10% roselle leaf extract group.

ANOVA test conducted in this research did not show significant differences in the percentage of wound healing among the groups (p=0.167). From a clinical assessment point of view, there were 2 rats in the 15% roselle leaf extract gel group with almost 100% wound healing percentage and 1 rat in the 10% roselle leaf extract gel group. In the other 3 treatment groups, there were no rats with almost 100% wound healed. Subsequently, although there were no statistical differences, clinically, the 15% roselle leaf extract gel treatment provided a faster healing effect in wounds.

Re-epithelialization is one of the important factors in wound closure and an important histological feature for ideal wound healing [20]. In the epithelialization process, keratinocytes are the main cellular components of the epidermis that are responsible for restoring the epidermis after injuries [21].

Growth factor (GF) is released in the wound healing process, triggering neovascularisation, repairing damaged blood vessels, and stimulating fibroblast proliferation and migration to wounds [6]. Fibroblast proliferation will produce a matrix that is important in the formation of a new extracellular matrix (ECM) and tissue repair [1]. Once the matrix is formed, keratinocytes will migrate and undergo proliferation [22] to cover wounds layer by layer and adhere to the underlying matrix [6]. Quercetin contained in roselle leaves [10] plays a role in wound healing by triggering the formation of ECM and increasing the synthesis of vascular endothelial growth factor (VEGF) and transforming growth factor (TGF-β) [15]. Research on quercetin topical in experimental animals showed that the compound modulates cytokines, GF, and cells involved in inflammatory and proliferative phases of wound healing. This activity leads to an increase in epithelial cell growth and indicates a faster wound healing process [16]. In addition, kaempferol, a bioflavonoid contained in roselle leaves [10], is proven to be correlated with VEGF and strengthen its angiogenic function, as well as having positive effects on keratinocyte cell migration, which is important in the re-epithelialization of wounds [23].

Histological assessment in this research showed that the higher the dose of roselle leaf extract, the greater the possibility of increased epithelization thickness in white rats. The rats in the group given 15% roselle leaf extract gel had an average of 64.69 µm epithelial thickness. Despite no statistically significant differences observed among the treatment groups in the epithelial growth, 15% roselle leaf extract gel showed a faster re-epithelialization process in wound healing. Moreover, based on clinical and histological assessment, the 10% and 15% roselle leaf extract gel treatment showed better wound healing processes than the positive control group.

To our knowledge, no study has been conducted on roselle leaf extract and wound healing. Studies were done on roselle but were limited to the use of their petals. The study done by Builder et al. on Wistar albino rats showed that roselle petal extract cream at 1.0%, 5%, and 10% concentration had significant wound healing activity according to the dose given [24]. In Indonesia, the research on roselle flower extract gel at 5%, 10%, and 15% concentration in rabbits showed the best wound healing effect at 15% roselle flower extract gel [25].

This research is a preliminary study to determine the effect of roselle leaf extract on wound healing. Overall, the study showed that the 15% roselle leaf extract gel provided faster wound healing in white rats based on clinical and histological assessment, despite no statistically significant differences among other treatment groups.

## Conclusion

This research showed the healing properties of roselle leaf extract gel, with a greater wound healing process in the 15% roselle leaf extract gel based on clinical and histological assessment. Although there were no statistically significant differences among the groups, roselle leaf extract brought forward the opportunity to be investigated further as a potential modality for wound healing.

## Personal thanks

We thank the Head of Dermatology and Venereology, Medical Faculty of Universitas Sumatera Utara and Universitas Sumatera Utara Hospital.
